# A pilot study of the functionality and clinician acceptance of a clinical decision support tool to improve primary care of opioid use disorder

**DOI:** 10.1186/s13722-021-00245-7

**Published:** 2021-06-15

**Authors:** Rebecca C. Rossom, JoAnn M. Sperl-Hillen, Patrick J. O’Connor, A. Lauren Crain, Laurel Nightingale, Anne Pylkas, Kristen V. Huntley, Gavin Bart

**Affiliations:** 1 HealthPartners Institute, Minneapolis, MN USA; 2 University of Minnesota School of Medicine, Minneapolis, MN USA; 3 HealthPartners Medical Group, Minneapolis, MN USA; 4 Sage Prairie Clinic, Eagan, MN USA; 5 Center for the Clinical Trials Network, National Institute on Drug Abuse, Bethesda, MA USA; 6 Hennepin Healthcare, Minneapolis, MN USA

**Keywords:** Opioid use disorder, Primary care, Clinical decision support

## Abstract

**Objective:**

Most Americans with opioid use disorder (OUD) do not receive indicated medical care. A clinical decision support (CDS) tool for primary care providers (PCPs) could address this treatment gap. Our primary objective was to build OUD-CDS tool and demonstrate its functionality and accuracy. Secondary objectives were to achieve high use and approval rates and improve PCP confidence in diagnosing and treating OUD.

**Methods:**

A convenience sample of 55 PCPs participated. Buprenorphine-waivered PCPs (n = 8) were assigned to the intervention. Non-waivered PCPs (n = 47) were randomized to intervention (n = 24) or control (n = 23). Intervention PCPs received access to the OUD-CDS, which alerted them to patients at potentially increased risk for OUD or overdose and guided diagnosis and treatment. Control PCPs provided care as usual.

**Results:**

The OUD-CDS was functional and accurate following extensive multi-phased testing. PCPs used the OUD-CDS in 5% of encounters with at-risk patients, far less than the goal of 60%. OUD screening confidence increased for all intervention PCPs and OUD diagnosis increased for non-waivered intervention PCPs. Most PCPs (65%) would recommend the OUD-CDS and found it helpful with screening for OUD and discussing and prescribing OUD medications.

**Discussion:**

PCPs generally liked the OUD-CDS, but use rates were low, suggesting the need to modify CDS design, implementation strategies and integration with existing primary care workflows.

**Conclusion:**

The OUD-CDS tool was functional and accurate, but PCP use rates were low. Despite low use, the OUD-CDS improved confidence in OUD screening, diagnosis and use of buprenorphine.

*NIH Trial registration* NCT03559179. Date of registration: 06/18/2018. URL: https://clinicaltrials.gov/ct2/show/NCT03559179

**Supplementary Information:**

The online version contains supplementary material available at 10.1186/s13722-021-00245-7.

## Introduction

The United States is in the midst of an opioid crisis [1]. Approximately 2 million Americans have opioid use disorder (OUD) [2], and over 49,000 people in the US died from opioid overdoses in 2019 [3]. Unfortunately, only 20% of patients with OUD seek treatment, and only 25% of those receive medications for OUD [4]. Ultimately, less than 10% of Americans with moderate-to-severe OUD receive treatment [2].

Primary care is the most common contact point for healthcare, and thus improving identification and treatment of OUD in primary care could help reduce this treatment gap. Buprenorphine (usually formulated in combination with naloxone) is an effective treatment for OUD in primary care [5–11], but only a fraction of primary care providers (PCPs) complete the required additional training to become “waivered” and eligible to prescribe buprenorphine. Of those who are waivered, over 70% never go on to prescribe buprenorphine [12]. Clinicians identify lack of staff training, institutional support, confidence, time, and access to clinical guidelines as barriers to prescribing buprenorphine [12–15]. Non-waivered PCPs have important roles to play in identifying patients with OUD and connecting them with treatment, but many do not feel comfortable doing so [15].

In the last decade, electronic health record (EHR)-linked web-based point-of-care clinical decision support (CDS) systems designed to improve primary care quality have become increasingly sophisticated and successful [16–20]. CDS tools can be particularly powerful tools when PCPs are less familiar with standards of care or when evidence-based care can be relatively complicated [21], both of which are true for OUD. However, to our knowledge, this type of CDS has not been implemented to improve primary care of OUD.

In this pilot study, we sought to take the first step towards an EHR-linked OUD-CDS tool based on the National Institute on Drug Abuse (NIDA) National Drug Abuse Treatment Clinical Trials Network (CTN) Dissemination Initiative’s white paper [22], which is based on national evidence-based guidelines [23, 24].

## Methods

### Setting

The study was conducted at HealthPartners, an integrated healthcare system that provides comprehensive medical care for over 1.2 million patients. At the time of this study, there were no systematic efforts to screen for OUD, and the primary process for treating OUD, if identified, was referral to specialty addiction clinics.

### Study oversight

The study protocol was approved by the HealthPartners Institutional Review Board, which approved a waiver of written consent for patients. The study was approved and monitored by the NIDA CTN Data and Safety Monitoring Board.

### Study design and participants

*Phase 1* (February 2017 to May 2018) aimed to develop a functional and accurate OUD-CDS tool and did not involve study participants. *Phase 2* (June to December 2018) pilot tested the clinical content, interface format, and usability of the OUD-CDS with a convenience sample of volunteer PCPs recruited via emailed invitations. Eligible PCPs (1) were primary care physicians or advanced practice providers (APPs) engaged in independent primary care of adults, (2) had at least schedule 3 Drug and Enforcement Agency prescribing privileges, (3) had at least half-time clinical primary care responsibilities, and (4) provided informed consent. PCPs were offered gift cards to participate in the study and complete baseline and 6-month surveys.

### Randomization

Recruited PCPs who were buprenorphine-waivered (“waivered”) were given OUD-CDS access. PCPs who were not buprenorphine-waivered (“non-waivered”) were randomized equally to receive or not receive OUD-CDS access within strata defined by provider type (physician, APP) and proportion of patient panel with three or more opioid prescriptions (above, below median). All PCPs were assigned a random number using a random number generator and seed based on the randomization date (20180529).

### Study procedures

*Phase 1* employed a highly iterative process to develop the OUD-CDS. In *Phase 1a*, the NIDA CTN Dissemination Initiative’s white paper [22] was deconstructed and rebuilt into web-based algorithms suitable for the OUD-CDS tool. Investigators identified the medications, comorbidities, laboratory results and other data needed to construct the OUD-CDS algorithms. Web and EHR programmers determined EHR data elements that could be harvested to inform the algorithms, how to process these algorithmically, and how to display output within the CDS tool. In sum, the seven pages of dense algorithms from the white paper became 87 separate algorithms.

In *Phase 1b*, multiple iterations of the user interface were developed—first in wireframes, then on a web platform, and finally as a web platform integrated with the EHR. Detailed feedback was solicited from study team members and physicians throughout this process.

In *Phase 1c*, two primary and three addiction medicine physician investigators tested the OUD-CDS in the EHR testing environment using a library of test patients developed to represent various relevant clinical scenarios.

Finally, in *Phase 1d*, the CDS was programmed to function silently in the EHR production environment for all real office encounters in without displaying to the end user. For patients meeting eligibility criteria, the CDS that would have displayed at each encounter was validated by testing clinicians using chart audit abstraction to confirm the accuracy and appropriateness of the CDS. Where indicated, this was followed by programming changes and iterative testing to ensure resolution.

In *Phase 2*, a convenience sample of PCPs was recruited. PCPs assigned (waivered) or randomized (non-waivered) to receive the intervention that identified people at risk for OUD at office encounters and recommended use of the OUD-CDS. These clinicians were emailed a link to an online training session prior to the intervention’s start. The training provided step-by-step instructions for the OUD-CDS and reinforced the importance of OUD diagnosis and treatment in primary care; all intervention clinicians completed the training. Intervention PCPs were asked to provide feedback on the OUD-CDS via a “Suggestions” tab on the CDS display. Additionally, four intervention PCPs were informally interviewed to gather information to improve the design of the OUD-CDS.

### Outcomes

The *primary outcome* was to demonstrate that the OUD-CDS tool was functional and accurate. In the second phase, the CDS output was provided to PCPs to assess *secondary outcomes* of OUD-CDS use rates, PCP confidence in diagnosing and treating OUD, and likelihood of PCPs to recommend the OUD-CDS to colleagues. *Exploratory outcomes* included rates of OUD diagnosis, prescriptions of medications for OUD and referrals for OUD treatment.

#### Study measures

Use rates of the CDS tool were captured via the web service. At baseline, PCPs completed 15-item surveys assessing confidence recognizing and treating patients with OUD. At the end of the intervention, all PCPs completed questions re-assessing confidence, and intervention PCPs completed questions about their experience with the OUD-CDS (Additional file 1: Appendix A). Data on exploratory outcomes, such as rates of OUD diagnosis or naloxone prescriptions, were collected from the EHR in the 3 months prior to and the 6 months of the intervention.

### OUD-CDS tool

The OUD-CDS identified patients at potentially elevated risk for OUD or opioid overdose at office encounters and displayed an alert banner within the EHR inviting PCPs to open the OUD-CDS. The alert displayed for patients: (1) at increased risk for OUD due to having 3 or more opioid prescriptions in the last year or an opioid prescription and a diagnosis of substance use disorder other than OUD or nicotine, (2) with an OUD diagnosis, or (3) with a prescription or documented use of buprenorphine or methadone (naltrexone was not included because the vast majority of naltrexone was prescribed for alcohol use disorder). These criteria resulted in the banner displaying at 14% of all adult primary care encounters. This was deemed too high of a burden on PCPs by the study team, as the prevalence rate of OUD in primary care clinics was likely a fraction of that based on national data [25], so the criterion of having an opioid prescription plus a diagnosis of non-OUD/nicotine substance use disorder was dropped, resulting in the EHR banner displaying at 8% of primary care encounters.

**Fig. 1:**
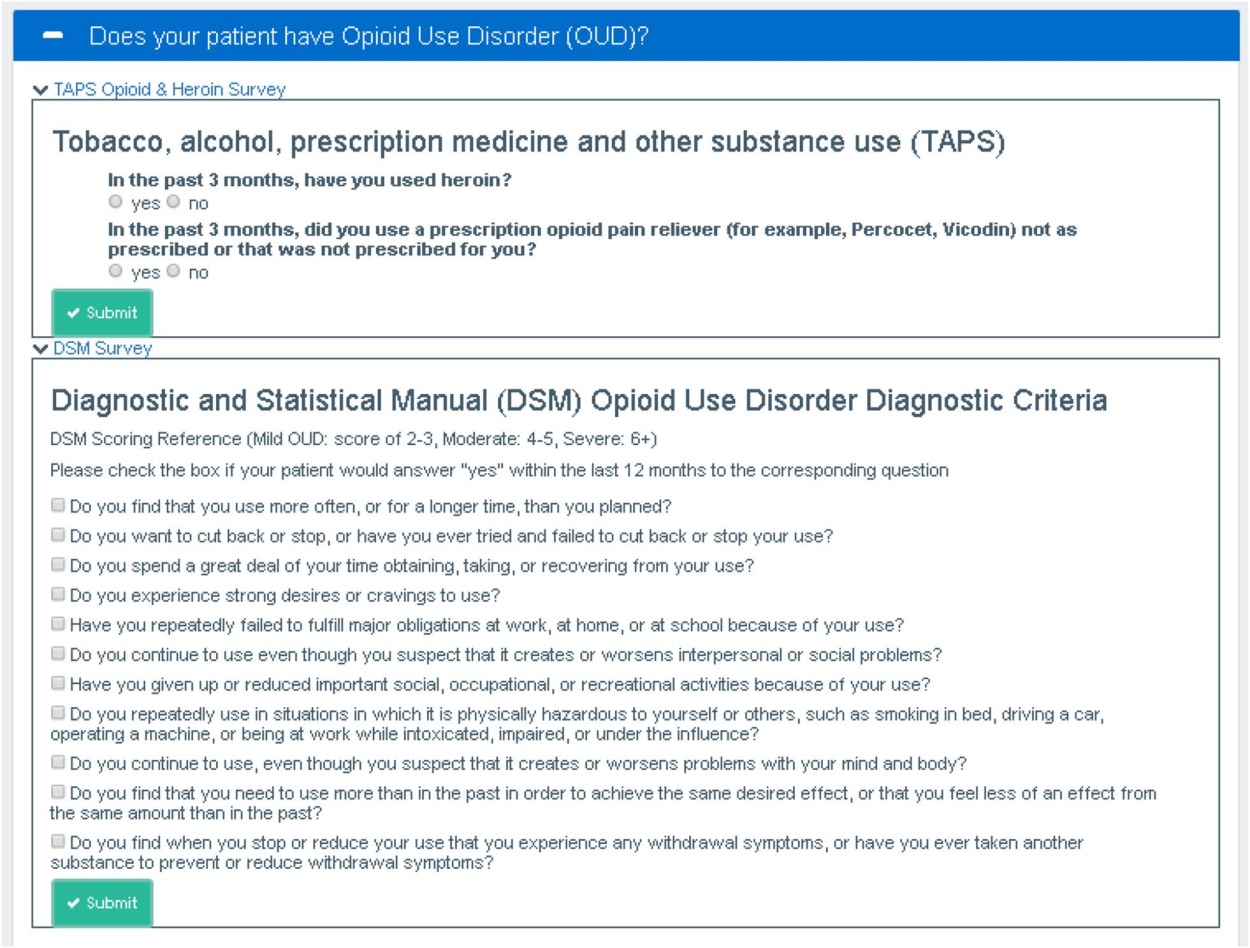
Screening and diagnosis module

The OUD-CDS included the following modules:

The Screening and Diagnosis Module (Fig. 1) provided screening questions for OUD using the opioid and heroin items from the TAPS (Tobacco, Alcohol, Prescription Medication and Other Substance Use Tool) [26]. It also provided a checklist of the OUD diagnostic criteria from the DSM-5 (Diagnostic and Statistical Manual of Mental Disorders) [27] and provided access to the state Prescription Drug Monitoring Database (PDMP).

**Fig. 2:**
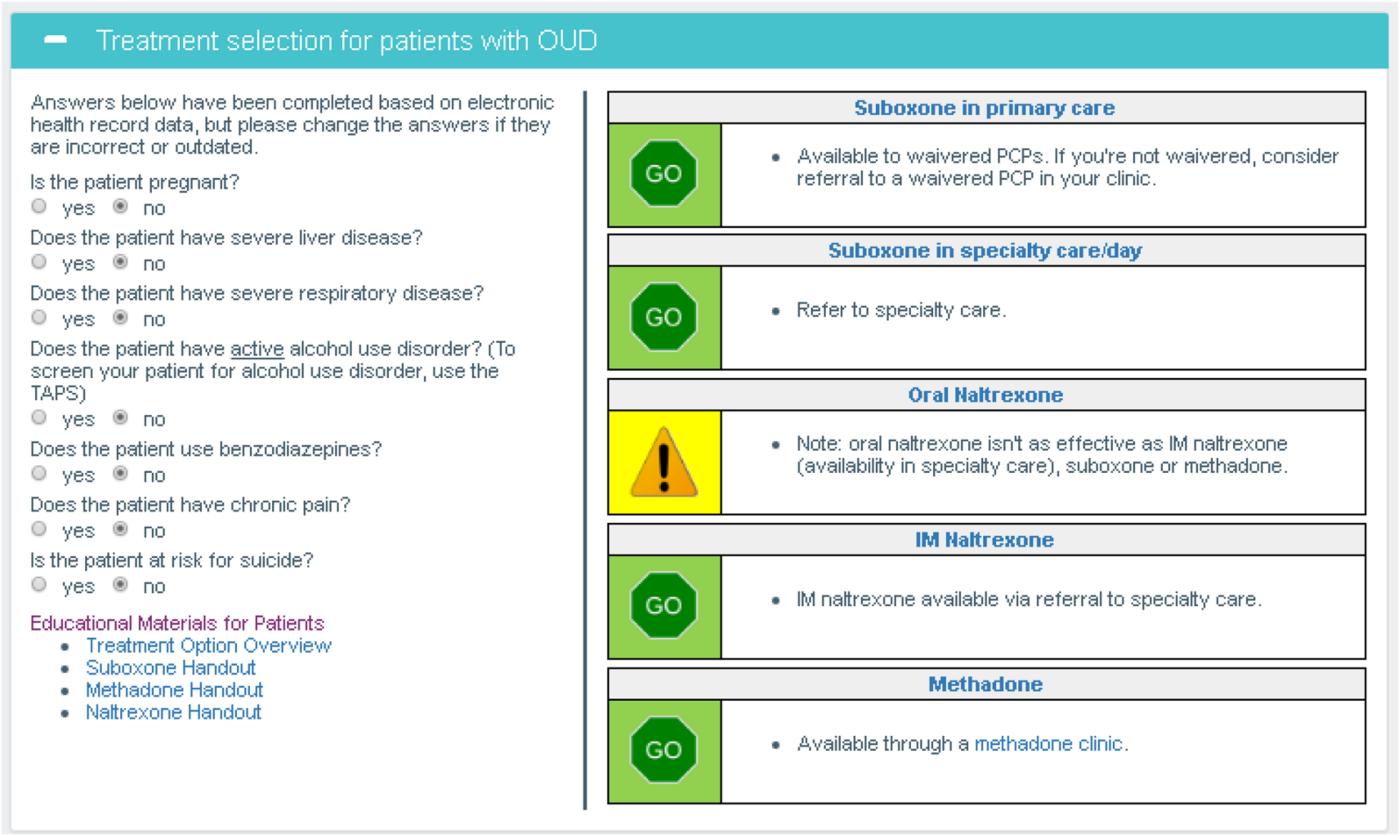
Treatment selection module

The Treatment Selection Module (Fig. 2) included radio buttons pre-populated with EHR data about relative contraindications to OUD medications or treatment in primary care. PCPs were able to change these radio buttons if they were aware of updated clinical information that was not in the EHR, such as pregnancy, which changed the treatment recommendations in real time. Non-waivered PCPs could use the tool to refer patients wanting buprenorphine to specialty care and to screen for and treat comorbid mental health conditions or infectious diseases.

**Fig. 3:**
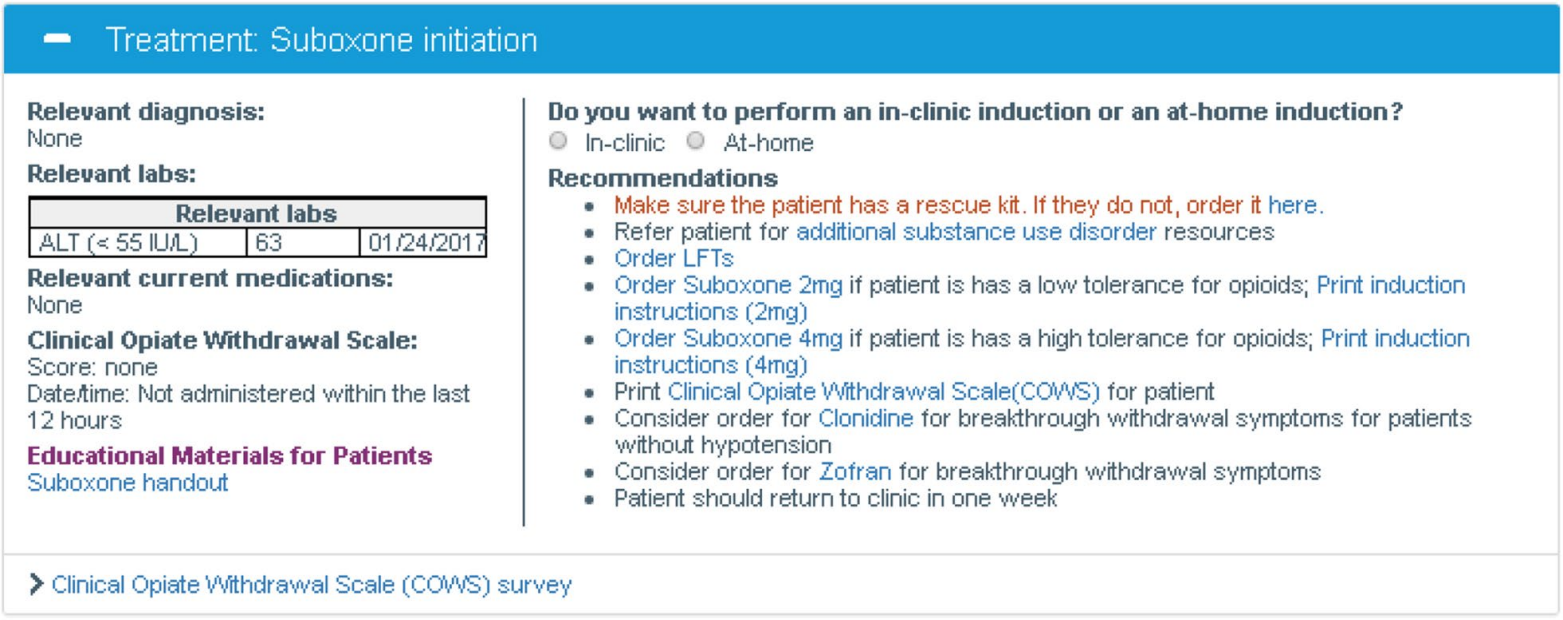
Suboxone (buprenorphine plus naloxone) initiation module

The OUD-CDS included several Treatment Initiation and Maintenance Modules:

- The Buprenorphine Initiation Module (Fig. 3) provided decision support for either in-office or at-home buprenorphine induction. Hyperlinks in the OUD-CDS populated orders into the EHR in a pop-up box that accumulated orders for the PCP to later review, edit and delete or sign. The OUD-CDS provided hyperlinked orders for buprenorphine initiation at two starting doses, accompanied by detailed handouts for patients describing how and when to start this medication. It also provided access to and guidance on how to use the COWS (Clinical Opiate Withdrawal Scale) [28]. The module included hyperlinked orders for recommended laboratory tests and adjuvant as-needed medications (e.g., clonidine and ondansetron) to manage withdrawal symptoms. It also included a hyperlinked referral for behavioral health care managers to assess patients’ needs for therapy and other substance use disorder or mental health services.
- The Buprenorphine Maintenance Module guided PCPs in assessing effectiveness and adherence, adjusting the buprenorphine dose if indicated, and obtaining a urine toxicology screen.
- The Naltrexone Initiation module was limited to oral naltrexone because PCPs did not prescribe intramuscular naltrexone due to billing complexities. However, it should be noted that oral naltrexone is not indicated for the treatment of OUD [29]. The module provided guidance on assessing social supports to determine whether the patient should have daily or thrice weekly dosing, stressed the importance of the patient being fully withdrawn from opioids prior to initiation, provided access and guidance to use of the COWS, provided hyperlinked orders for baseline and 12-week liver function tests, and provided a hyperlinked referral for a behavioral health care assessment.
- The Naltrexone Maintenance Module helped PCPs assess effectiveness and adherence, adjust the naltrexone dose or consider other OUD medications if indicated, and obtain a urine toxicology screen.
- The Methadone Module provided medication information, offered a printable list of community clinics offering methadone treatment, and guided PCPs in assessing methadone adherence.

**Fig. 4:**
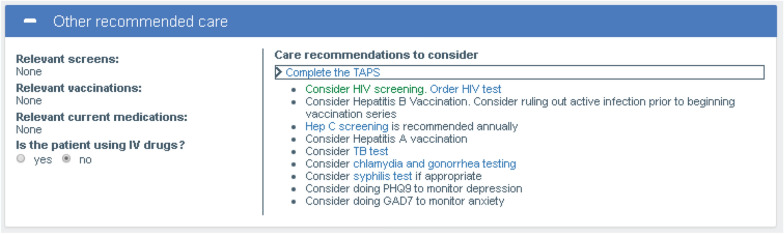
Other recommended care module

The Other Recommended Care Module (Fig. 4) guided PCPs in screening and/or vaccinating for infectious diseases, screening for depression, suicidal ideation and anxiety, and offering HIV pre-exposure prophylaxis and a handout on safer injection drug use for patients using drugs by injection.

### Statistical analysis

Average use rates across all intervention PCPs were calculated for each month of the intervention period. A goal use rate of 60% was chosen because this would reflect use in the majority of visits in which it was indicated while allowing for some visits where another clinical concern would take priority over OUD screening. Changes in PCP survey ratings were assessed via linear mixed models in which repeated survey ratings were predicted from survey timing (post- vs. pre-intervention), PCP group (waivered CDS access, non-waivered CDS access, non-waivered no CDS access) and the timing by PCP group interaction. Post- versus pre-intervention contrasts within each PCP group tested whether PCP ratings changed significantly over time (p < 0.05). Rate ratios for each exploratory outcome were calculated separately by PCP group as the post-intervention relative to pre-intervention rates per patient-year to describe the extent to which PCPs changed the frequency with which they diagnosed OUD, ordered OUD medications or referred patients for OUD treatment.

## Results

### Study population

Fifty-five PCPs participated in the study, including 32 physicians, 17 physician assistants, and 6 nurse practitioners (Table 1). The 8 waivered PCPs were all physicians and assigned to receive OUD-CDS access. The remaining 47 non-waivered PCPs were randomized to receive (n = 24) or not receive (n = 23) OUD-CDS access. PCPs tended to be non-Hispanic white (76%) female (60%) family medicine (65%) physicians (60%). During the 6-month pilot intervention, 5199 primary care patients (3220 intervention, 1979 control) made 8304 study-eligible visits (5153 intervention, 3151 control). In all, 2998 patients made more than one visit during this period.

**Table 1:** Demographic characteristics of N = 55 randomized PCPs

		All	Control n = 23	Intervention non-waivered n = 24	Intervention waivered n = 8
Provider type					
Physician	%	60	52	54	100
Nurse practitioner	%	11	13	13	0
Physician Assistant	%	29	35	33	0
Medical specialty					
Family practice	%	66	74	58	63
Internal medicine	%	29	22	33	38
Med Peds	%	6	4	8	0
Sex					
F	%	60	70	54	50
M	%	40	30	46	50
Age	Mean	43	42	43	46
Hispanic ethnicity					
Yes	%	2	0	0	13
Race					
White	%	76	78	79	63
Black	%	6	4	4	13
Asian	%	11	9	17	0
Multiple	%	4	4	0	13
Unknown	%	4	4	0	13
Years in practice following residency or fellowship					
0–5	%	26	35	21	13
6–10	%	24	26	25	13
11–15	%	16	9	17	38
16–20	%	18	13	25	13
21+	%	16	17	13	25

### Primary outcome: function and accuracy of the OUD-CDS

Testing in the EHR and chart audit validation demonstrated that the OUD-CDS tool was functional and accurate. In assessments with 83 simulated patients in the EHR test environment and in expert and primary care clinician chart reviews of 165 real patients at various levels of risk for OUD or with diagnosed OUD, radio buttons were found to be accurately pre-populated and OUD medication and other treatment suggestions were deemed clinically accurate and guideline-concordant. Additionally, during Phase 2, there were no concerns about the OUD-CDS output reported by PCPs.

### Secondary outcomes: PCP use rates, confidence in diagnosing and treating OUD, and likelihood to recommend

The alert banner for encounters with at-risk patients displayed for 3220 patient encounters with intervention PCPs, who on average opened the OUD-CDS tool for 5.05% of eligible encounters, far less than the goal of 60% (Table 2). OUD-CDS use rates by individual PCPs ranged from 0 to 40% of eligible patient visits. The most used CDS components were the opioid questions from the TAPS (98%), the non-opioid questions from the TAPS (22%), the OUD DSM criteria (15%) and the link to the PDMP (11%). Percent use of other components was in the single digits. PCPs ordered naloxone in 6% of encounters.

**Table 2:** Monthly PCP use rates of the OUD-CDS for targeted higher-risk patient encounters among intervention PCPs

	n PCPs who had a banner display (i.e., had 1+ study eligible visit)			n study eligible visits			% of study eligible visits where the OUD-CDS was opened		
	All	Waivered		All	Waivered		All	Waivered	
		No	Yes		No	Yes		No	Yes
June 2018	32	24	8	642	437	205	8.90	9.52	7.04
July 2018	32	24	8	667	458	209	3.35	3.58	2.70
Aug 2018	31	23	8	667	450	217	3.02	3.64	1.23
Sept 2018	31	23	8	508	336	172	6.67	7.40	4.57
Oct 2018	30	22	8	397	233	164	5.25	6.01	3.17
Nov 2018	29	21	8	339	197	142	2.14	1.19	4.65
Total	32	24	8	3220	2111	1109	5.05	5.44	3.87

**Fig. 5:**
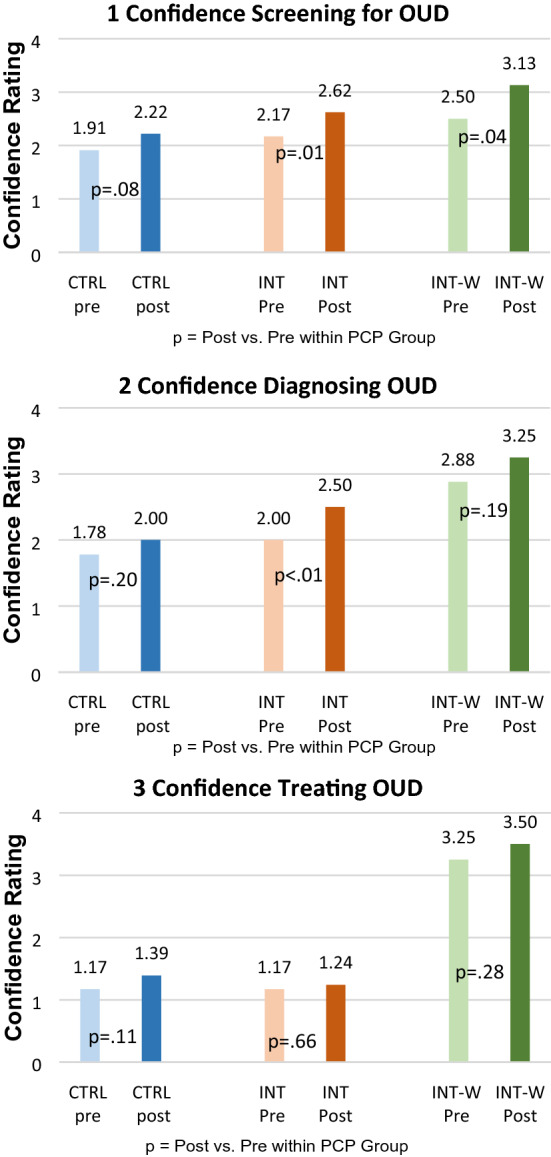
PCP confidence in screening, diagnosing and treating OUD. *INT* intervention non-waivered PCPs, *INT-W* intervention waivered PCPs

All PCPs completed baseline surveys and 98% completed 6-month surveys. Although this pilot study did not entail power calculations or pre-specify statistical endpoints, exploratory statistical analyses revealed that despite low use rates, intervention PCPs reported increased confidence in screening for OUD at the end of the pilot compared to pre-pilot confidence on 4-point Likert scale questions (waivered: 3.13 vs. 2.50, p < 0.04; non-waivered: 2.62 vs. 2.17, p < 0.02; Fig. 5), while control PCPs did not (2.22 vs. 1.91, p < 0.09). Confidence in diagnosing OUD increased significantly for non-waivered intervention PCPs (2.50 vs. 2.00, p < 0.01), but not for waivered intervention PCPs (3.25 vs 2.88, p < 0.19) or control PCPs (2.00 vs 1.78, p < 0.20). There were no changes in confidence in treating patients with OUD in any group.

At the end of the intervention, 75% of waivered PCPs and 62% of non-waivered PCPs were moderately or very likely to recommend the OUD-CDS (Table 3). In all, 89% reported that the OUD-CDS helped them screen for OUD, 86% that it made them feel more comfortable prescribing OUD medications, 93% that it made it easier to discuss OUD treatment options with patients and determine their preferences, and 93% that it helped them know when to refer patients for specialty treatment. The majority of PCPs with OUD-CDS access felt the following features of the tool were moderately or very useful: prescribing naloxone (89%), deciding which treatment approach is best for patients (78%), receiving safety alerts for drug–drug interactions (93%), and receiving urine drug screen reminders (93%).

**Table 3:** PCP ratings of the OUD-CDS among intervention PCPs at the end of the 6-month intervention

		All	Intervention waivered	
		n = 32	No n = 24	Yes N = 8
How likely are you to recommend Opioid Wizard to a colleague?	Moderately or very	66%	62%	75%
Opioid Wizard is a tool that helps me screen for OUD	Somewhat or strongly agree	89%	95%	75%
Opioid Wizard makes me feel more comfortable prescribing medications for OUD in practice	Somewhat or strongly agree	86%	85%	88%
Opioid Wizard makes it easier to discuss treatment options of OUD with patients and determine their preference	Somewhat or strongly agree	93%	95%	88%
Opioid Wizard helps me know when to refer patients for methadone or other specialty treatment	Somewhat or strongly agree	93%	100%	75%
How useful are the following Opioid Wizard features?				
Prescribing overdose kits	Moderately or very	89%	90%	86%
Deciding which treatment approach is best for the patient	Moderately or very	78%	75%	86%
Safety alerts for drug-drug interactions	Moderately or very	93%	95%	86%
Urine drug screen testing reminders	Moderately or very	93%	90%	100%

### Exploratory outcomes: post- vs. pre-intervention comparisons of OUD diagnosis, OUD medication prescriptions and referrals

Table 4 shows that rates of OUD diagnosis for higher-risk patients increased 28.5-fold for non-waivered intervention PCPs and 11.3-fold in waivered intervention PCPs compared to 7.9-fold in control PCPs. No higher-risk patients started buprenorphine pre-intervention, and no higher-risk patients of control PCPs started buprenorphine during the intervention, but higher-risk patients of waivered and non-waivered intervention PCPs started buprenorphine at low rates during the intervention. Rates of buprenorphine initiation for patients with diagnosed OUD increased by 40% in waivered intervention PCPs, with smaller increases for non-waivered intervention PCPs (10%) and control PCPs (3%). Naltrexone initiation was low across PCP groups. Naloxone was also prescribed at relatively low rates and generally declined during the intervention for higher-risk patients and patients with known OUD, but did increase 18% for higher risk patients of control PCPs. Referrals for OUD treatment generally increased for all higher-risk and OUD-diagnosed patients during the intervention.

**Table 4:** Exploratory outcomes (presented as rates per patient-year)

Outcome	Patient sample	Period	Control PCPs	Intervention PCPs Waivered	
			n = 23	No n = 24	Yes n = 8
OUD diagnosis	Higher risk for OUD	Pre	0.008	0.005	0.013
		Post	0.062	0.149	0.152
		Post/pre	7.87	28.54	11.30
Buprenorphine starts	Higher risk for OUD	Pre	0	0	0
		Post	0	0.029	0.015
		Post/pre	NA	NA	NA
Buprenorphine starts	Diagnosed with OUD	Pre	0.359	0.566	3.666
		Post	0.368	0.625	5.127
		Post/pre	1.03	1.10	1.40
Naltrexone starts	Higher risk for OUD	Pre	0	0	0
		Post	0.030	0.019	0.041
		Post/pre	NA	NA	NA
Naltrexone starts	Diagnosed with OUD	Pre	0.049	0.049	0.053
		Post	0.020	0.074	0.045
		Post/pre	0.42	1.52	0.85
Naloxone starts	Higher risk for OUD	Pre	0.133	0.176	0.256
		Post	0.157	0.166	0.122
		Post/pre	1.18	0.95	0.48
Naloxone starts	Diagnosed with OUD	Pre	0.310	0.486	0.481
		Post	0.286	0.294	0.295
		Post/pre	0.92	0.61	0.61
Referrals to specialty care	Higher risk for OUD	Pre	0.106	0.101	0.103
		Post	0.149	0.130	0.162
		Post/pre	1.41	1.29	1.57
Referrals to specialty care	Diagnosed with OUD	Pre	0.238	0.205	0.283
		Post	0.247	0.247	0.281
		Post/pre	1.04	1.21	0.99

## Discussion

Through a highly iterative testing process, the OUD-CDS was found to be functional and accurate, and the majority of PCPs reported that they would recommend the tool to colleagues. However, OUD-CDS use rates were much lower than targeted for visits with at-risk patients. Notwithstanding low use, improvements were seen in self-reported confidence in screening for OUD for all intervention PCPs and in diagnosing OUD for non-waivered intervention PCPs. The OUD-CDS did not impact confidence in OUD treatment.

Despite PCPs generally liking the tool, use rates were disappointingly low. Across many, if not most, studies of clinical CDS tools, the main barriers have been adoption and use, and are primary reasons why numerous CDS implementation studies for diabetes and other chronic disease have failed [30, 31]. Ultimately, use rates have been higher when rooming staff (rather than clinicians) have triggered the CDS, and when the CDS has been available to both the patient and clinician [32]. We think there were multiple contributors to low use in our pilot. First, because of its small sample size and short duration, we were unable to implement our usual practice of conducting clinic-randomized studies where a best practice advisory displays to rooming staff, allowing them to print the CDS and give paper versions to patients and PCPs. In studies where we engage rooming staff in the workflow, use rates have ranged from 71 to 77% [33]. Second, in informal interviews, PCPs reported not seeing the alert banners in the EHR, and we subsequently learned, to the surprise of the healthcare system, that PCPs were able to move this alert section to the bottom of their EHR display where it went unseen. Third, again in informal interviews, PCPs did not feel that they had adequate time to address opioid risk or felt that OUD was a low priority for particular patients or encounters. Fourth, the OUD-CDS may have been too complicated for use in primary care, with some PCPs stating they would rather have an “easy button” that would refer any at-risk patients to specialty care. Finally, we displayed the OUD-CDS banner at 8% of all adult primary care visits, meaning most flagged patients did not have OUD; this relatively low positive predictive value may have led to decreasing use of the OUD-CDS over time.

Despite the iterative designs and expert input from clinicians, informal interviews provided feedback that the tool would be more useful if it were more intuitive and easier to use, especially for PCPs less experienced with OUD. PCPs were not always sure when they were “done” with the OUD-CDS, and at times seemed overwhelmed by the complexity of comprehensive and non-prioritized OUD care recommendations. This feedback has led our team to extensively redesign the OUD-CDS, making the tool simpler and more modular while also maintaining flexibility for more experienced users. We are also considering reducing the proportion of patients targeted for the OUD-CDS alert by using more sophisticated and more accurate EHR-based risk algorithms.

In addition to planning changes to the interface, we also anticipate adjusting implementation of the OUD-CDS to: (a) Incorporate the OUD-CDS into an integrated platform with other CDS tools that are being utilized successfully at high rates at office visits, (b) Have rooming staff trigger and support interaction with the OUD-CDS, (c) Provide clinic leaders and clinical teams with feedback about CDS use rates, and (d) Deploy problem-solving strategies to improve CDS use rates for specific clinics or care teams when necessary. These changes will be incorporated in a clinic-randomized trial across three healthcare systems (ClinicalTrials.gov Identifier: NCT04198428).

We have discussed several potential limitations to this pilot study, including low use of the tool by PCPs, leading to a more limited ability to understand usability and generalizability. We did not survey PCPs as to why they did not frequently use the tool, and only have this information from informal PCP interviews, an additional limitation. Another limitation may have been providing training only to intervention PCPs, potentially making PCPs more comfortable with OUD care regardless of their level of interaction with the OUD-CDS; however passive didactic training alone does not tend to impact clinical outcomes [34]. An additional limitation is that we were unable to determine the accuracy of the algorithms that alerted clinicians to patients who were potentially at-risk for OUD. Relatedly, our risk algorithms were only able to take opioid prescriptions documented in the EHR into account. While we would have liked to have included opioid prescriptions documented in the PDMP, we were not able to obtain permission to upload and manipulate these data from the vendor who holds the state contract for electronic access to these data. Finally, our study was conducted in a highly integrated care system, which may limit the study’s generalizability to other settings.

## Conclusions

The OUD-CDS was functional and accurate and effected changes in PCP confidence in screening and diagnosing OUD despite low use rates. Most PCPs recommended the tool and found it helpful with key aspects of OUD care. A large multi-site study is in progress that will incorporate PCP feedback to make the OUD-CDS more intuitive and simple and will include implementation strategies to achieve higher CDS use, including tapping into established rooming staff workflows.

## Supplementary Information

**Additional file 1: Appendix A. **Baseline Primary Care Clinician Survey.

## Data Availability

The datasets used and/or analyzed during the current study are available from the corresponding author on reasonable request.
