# Supplementary material for: A pilot study of the functionality and clinician acceptance of a clinical decision support tool to improve primary care of opioid use disorder
Source: Addict Sci Clin Pract. 2021 Jun 15;16:37. doi: 10.1186/s13722-021-00245-7 (PMC8207778; doi:10.1186/s13722-021-00245-7)
Supplement: Supplementary file 1 — Additional file 1: Appendix A. Baseline Primary Care Clinician Survey. [file 13722_2021_245_MOESM1_ESM.docx]

**Baseline Primary Care Clinician Survey**

**Please answer the following questions to the best of your ability. Your answers will help us understand the impact Opioid Wizard may have on clinician ability to diagnose and treat opioid use disorder, and the usefulness of the tool.**

1. What is your primary clinic?
2. What is your age?
3. What is your gender
4. With which race/ethnicity do you identify?

- Asian
- Hispanic
- Native American/Alaskan Native
- Non-Hispanic black
- Pacific Islander/Native Hawaiian
- White
- Mixed race or other
- Prefer not to answer

1. How many years have you been in practice following residency or fellowship?

- 0-5
- 6-10
- 11-15
- 16-20
- 21+

1. What is your medical specialty?
   - Family Practice
   - Internal Medicine
   - Med Peds
   - Other (please specify)
2. On average, how many days a week do you see patients in clinic?
   - 0
   - 1
   - 2
   - 3
   - 4
   - 5 or more

Answer the questions below about your knowledge and approach to management of patients with Opioid Use Disorder (OUD). Choose only one answer.

1. How often do you formally assess patients for opioid use disorder (OUD)?

| Very often | Often | Sometimes | Occasionally | Never |
| --- | --- | --- | --- | --- |

1. How confident do you feel about screening your patients for OUD?

| Very confident | Moderately confident | Somewhat confident | Not at all confident |
| --- | --- | --- | --- |

1. How often do you provide treatment or refer your patients for treatment of OUD?

| Very often | Often | Sometimes | Occasionally | Never |
| --- | --- | --- | --- | --- |

1. How confident are you at diagnosing patients with OUD?

| Very confident | Moderately confident | Somewhat confident | Not at all confident |
| --- | --- | --- | --- |

1. How confident are you at treating your patients with medications such as buprenorphine or naltrexone for OUD?

| Very confident | Moderately confident | Somewhat confident | Not at all confident |
| --- | --- | --- | --- |

1. How confident are you at knowing when to refer your patients with OUD for treatment by addiction specialists?

| Very confident | Moderately confident | Somewhat confident | Not at all confident |
| --- | --- | --- | --- |

1. (Skip if certified in buprenorphine prescribing) To what extent do you agree or disagree with the following statement: The availability of EHR-integrated clinical decision support for how to use buprenorphine and/or naloxone and manage OUD would make it more likely that I would become a certified buprenorphine prescriber.

| Strongly agree | Somewhat Agree | Somewhat Disagree | Strongly disagree |
| --- | --- | --- | --- |

1. Please rate your ability to effectively manage patients with the following treatment strategies:

|  | High ability (on par with subspecialists) | Moderately high ability | Adequate ability | Some ability; could use improvement | Low  ability |
| --- | --- | --- | --- | --- | --- |
| Brief motivational counseling | 1 | 2 | 3 | 4 | 5 |
| Overdose prevention | 1 | 2 | 3 | 4 | 5 |
| Extended-release naltrexone (Vivitrol) | 1 | 2 | 3 | 4 | 5 |
| Buprenorphine (as monotherapy or in combination with naloxone (Suboxone)) | 1 | 2 | 3 | 4 | 5 |
| Referral for methadone or other treatment | 1 | 2 | 3 | 4 | 5 |

**6-month Primary Care Clinician Survey**

Answer the questions below about your knowledge and approach to management of patients with Opioid Use Disorder (OUD). Choose only one answer.

1. How often do you formally assess patients for opioid use disorder (OUD)?

| Very often | Often | Sometimes | Occasionally | Never |
| --- | --- | --- | --- | --- |

1. How confident do you feel about screening your patients for OUD?

| Very confident | Moderately confident | Somewhat confident | Not at all confident |
| --- | --- | --- | --- |

1. How often do you provide treatment or refer your patients for treatment of OUD?

| Very often | Often | Sometimes | Occasionally | Never |
| --- | --- | --- | --- | --- |

1. How confident are you at diagnosing patients with OUD?

| Very confident | Moderately confident | Somewhat confident | Not at all confident |
| --- | --- | --- | --- |

1. How confident are you at treating your patients with medications such as buprenorphine or naltrexone for OUD?

| Very confident | Moderately confident | Somewhat confident | Not at all confident |
| --- | --- | --- | --- |

1. How confident are you at knowing when to refer your patients with OUD for treatment by addiction specialists?

| Very confident | Moderately confident | Somewhat confident | Not at all confident |
| --- | --- | --- | --- |

1. (Skip if certified in buprenorphine prescribing) To what extent do you agree or disagree with the following statement: The availability of EHR-integrated clinical decision support for how to use buprenorphine and/or naloxone and manage OUD would make it more likely that I would become a certified buprenorphine prescriber.

| Strongly agree | Agree | Disagree | Strongly disagree |
| --- | --- | --- | --- |

1. Please rate your ability to effectively manage patients with the following treatment strategies:

|  | High ability (on par with subspecialists) | Moderately high ability | Adequate ability | Some ability; could use improvement | Low  ability |
| --- | --- | --- | --- | --- | --- |
| Brief motivational counseling | 1 | 2 | 3 | 4 | 5 |
| Overdose prevention | 1 | 2 | 3 | 4 | 5 |
| Extended-release naltrexone (Vivitrol) | 1 | 2 | 3 | 4 | 5 |
| Buprenorphine (as monotherapy or in combination with naloxone (Suboxone)) | 1 | 2 | 3 | 4 | 5 |
| Referral for methadone or other treatment | 1 | 2 | 3 | 4 | 5 |

**NOTE: **These questions are only be present in the post (6-month) surveys for primary care clinicians with CDS access****

Please answer the questions below concerning work flow and ease of use of the Opioid Wizard Clinical Decision Support.

1. How likely are you to recommend Opioid Wizard to a colleague?

| Very  likely | Moderately  likely | Somewhat likely | Not very likely | Not at all likely |
| --- | --- | --- | --- | --- |

1. Opioid Wizard is a tool that helps me screen for OUD.

| Strongly agree | Somewhat Agree | Somewhat Disagree | Strongly disagree |
| --- | --- | --- | --- |

1. Opioid Wizard makes me feel more comfortable prescribing medications for OUD in my practice.

| Strongly agree | Somewhat Agree | Somewhat Disagree | Strongly disagree |
| --- | --- | --- | --- |

1. Using the Opioid Wizard makes it easier for me to discuss treatment options of OUD with patients and determine their preference.

| Strongly agree | Somewhat Agree | Somewhat Disagree | Strongly disagree |
| --- | --- | --- | --- |

1. Opioid Wizard helps me know when to refer patients for methadone or other specialty treatment.

| Strongly agree | Somewhat Agree | Somewhat Disagree | Strongly disagree |
| --- | --- | --- | --- |

1. When I want or need to address OUD with patients, the Opioid Wizard saves me time.

| Strongly agree | Somewhat Agree | Somewhat Disagree | Strongly disagree |
| --- | --- | --- | --- |

1. Opioid Wizard improves my office efficiency.

| Strongly agree | Somewhat Agree | Somewhat Disagree | Strongly disagree |
| --- | --- | --- | --- |

1. Time using the Opioid Wizard with patients is time well spent.

| Strongly agree | Somewhat Agree | Somewhat Disagree | Strongly disagree |
| --- | --- | --- | --- |

1. Opioid Wizard influences my treatment recommendations.

| Strongly agree | Somewhat Agree | Somewhat Disagree | Strongly disagree |
| --- | --- | --- | --- |

1. How useful are the following Opioid Wizard features?

| **Feature** | **Very useful** | **Moderately useful** | **Somewhat**  **useful** | **Slightly useful** | **Not at all useful** |
| --- | --- | --- | --- | --- | --- |
| Screening tools (TAPS) |  |  |  |  |  |
| Diagnosis tools |  |  |  |  |  |
| Prescribing overdose kits |  |  |  |  |  |
| Guidance for screening for comorbidities such as alcohol use disorder, hepatitis, pregnancy |  |  |  |  |  |
| Deciding which treatment approach is best for the patient (medication-assisted therapy by primary care, referral to an addiction specialists for medication-assisted therapy, safer use, further discussion, etc.) |  |  |  |  |  |
| Deciding between different strategies of medication-assisted therapy (naltrexone vs. buprenorphine vs. methadone) |  |  |  |  |  |
| Safety alerts for drug/drug interactions |  |  |  |  |  |
| Urine drug screen testing reminders |  |  |  |  |  |

1. How could we make Opioid Wizard more useful?

1. Do you have any other feedback you’d like to share, or ways in which you think Opioid Wizard could be improved?

Thank you for your participation. Your input is greatly appreciated.
